# Warm Season Grain Legume Landraces From the South of Europe for Germplasm Conservation and Genetic Improvement

**DOI:** 10.3389/fpls.2018.01524

**Published:** 2018-10-22

**Authors:** Antonio M. De Ron, Penelope J. Bebeli, Valeria Negri, Maria Carlota Vaz Patto, Pedro Revilla

**Affiliations:** ^1^Department of Genetics and Plant Breeding, Misión Biológica de Galicia, National Spanish Research Council (CSIC), Pontevedra, Spain; ^2^Laboratory of Plant Breeding and Biometry, Department of Crop Science, Agricultural University of Athens, Athens, Greece; ^3^Dipartimento di Scienze Agrarie, Alimentari e Ambientali, Università degli Studi di Perugia, Perugia, Italy; ^4^Instituto de Tecnologia Química e Biológica António Xavier, Universidade Nova de Lisboa, Oeiras, Portugal

**Keywords:** adaptation, diversity, breeding, populations, *Phaseolus*, physiology, plant genetic resources, *Vigna unguiculata*

## Abstract

Currently, there is a high concern from consumers regarding food quality, with emphasis on the origin of food sources. We here review the current situation of beans (*Phaseolus* spp.) and cowpea (*Vigna unguiculata* (L.) Walp.) landraces in the South of Europe focusing on morpho-agronomic and genetic diversity and physiological adaptation to the different agrosystems, including the symbiotic association with rhizobia. Despite the reduction in the production and consumption of grain legumes in Southern Europe, the variability of common bean, runner bean and cowpea landraces in this region is adequately preserved *ex situ* in germplasm banks and in breeder collections in Portugal, Spain, Italy and Greece; however, on-farm (*in situ*) conservation in isolated areas mainly in gardens and small fields for farmers own consumption and local markets is not guaranteed. This variability can be used for the genetic improvement of varieties, which will result in environmental-friendly improved legumes for a sustainable production in the South of Europe as well as in other regions of the World.

## Legume Landraces

Food legumes are an important component of human diet and life for their contribution as source of protein but also for their support to the environment sustainability through the biological symbiotic fixation of nitrogen, and the enhancement of the ecosystem services because some of them are bee pollinated (De Ron, 2015; Suso et al., 2016). In the South of Europe, beans and cowpea are relevant nutritional and environmental resources well adapted to their agrosystem that should be genetically preserved and improved for their efficient use.

Landraces are traditional crop varieties or populations growing in specific locations and constitute valuable sources for breeding purposes as basic genetic material to obtain improved elite varieties. Usually a landrace is a mixture of a number of distinct homozygous lines in the case of self-pollinating crops (common bean, *Phaseolus vulgaris* L. and cowpea, *Vigna unguiculata* (L.) Walp.) (Raggi et al., 2013). In the case of cross-pollinated crops (runner or scarlet bean *P. coccineus* L.), the landraces are populations with more heterozygous components (Newton et al., 2010). They are maintained by farmers according to their preferences and the adaptation to their environment in ecological key areas. Merging several definitions (Spataro and Negri, 2013), a landrace can be defined as a “variable population, which is identifiable and usually has a local name, lacks formal crop improvement, is characterized by a specific adaptation to the environmental conditions of the cultivation area (tolerant to the biotic and abiotic physiological stresses of that area) and is closely associated with the uses, knowledge, habits, dialects, and celebrations of the people who have developed and continue to grow it” (Negri et al., 2009). As such, they are a cultural and biological diversity heritage of value for present and future generations.

Common bean, runner or scarlet bean and cowpea are the warm season Mediterranean legumes included in this review. Common bean is the most important food legume for direct human consumption on a global scale (De Ron et al., 2016a), while runner bean has a more limited cultivation. Cowpea is extensively cultivated in tropical and subtropical areas in Africa (especially in the Sub-Saharan Africa, SSA) and the Americas (Central and South America), but has limited importance in Southern Europe and in North America (De Ron, 2015).

These legumes could be used for fresh and dry seeds and fresh pods and they play an important role in the healthy European Mediterranean diet. Recently, the role of beans and other food legumes in human diet refers not only to its high protein content but also to the functional properties of some components that could contribute to reduce risk of several serious diseases (Hangen and Bennink, 2003; Thompson et al., 2009; Vaz Patto et al., 2015). Recent trends on legume nutritional quality key factors focus on new strategies to enhance consumer acceptance and improve legume functional properties.

**FIGURE 1:**
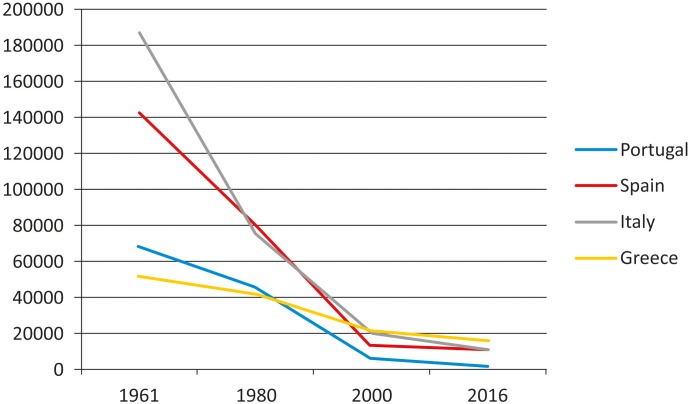
Production (Mg/year) evolution of dry bean (*Phaseolus vulgaris*) in southern European countries (FAOSTAT, 2018).

In spite of the decrease of grain legumes cultivation and consumption in some countries of Europe in the last years (Figure 1), the interest in landraces of these crops has recently grown in Europe, as well as in other continents This is due to the need of having a more sustainable agriculture, meet the present environmental challenges, avoid further genetic erosion and satisfy consumer increasing request for healthy, environmentally friendly, local food (with reduced physiological carbon footprint since it is locally produced). Special mention deserves the varieties that are recognized with some figure of legal protection, such as quality labels [like the European Protected Designation of Origin (PDO), Protected Geographical Indication (PGI), and Traditional Specialties Guaranteed (TSG)]. However, the wide variability and the lack of uniformity for many morpho-agronomic traits in landraces is an obstacle for the application of the current legislation for their commercial or protected registration in some countries.

## European Cowpea and Bean Landraces Evolution and Distinctness From the Original Genepools

Cowpea is the old “bean” that was grown by the Romans and Arabs (Álvarez de Morales, 2002). Domesticated in the SSA during the second millennium B.C., cowpea early spread in Asia and Europe, where it was grown by the Greeks in the third century B.C. and by the Romans in the first century based on the writings of Theophrastus and Plinius. With its spreading across the Old World, many different forms and landraces were developed also for this crop (Polegri and Negri, 2010).

Cowpea has been largely cultivated in the Old World, where this crop has a high cultural and socio-economic value for local communities (De Luca et al., 2018). Fresh pods of cowpea are consumed in Southern Europe, where a relatively large number of landraces has been developed, giving rise to a wide genotypic and phenotypic diversity among and within landraces (Lazaridi et al., 2017). These authors found that differences among cowpea landraces are not determined by the country of origin in Southern Europe. Neighbor landraces can be adequately distinguished even though there is a high level of diversity present within each landrace; consequently, the best strategy for maintaining diversity in an area is to preserve each of the landraces in the farms from which it came (Tosti and Negri, 2005).

After the initial domestication process in the Americas, the common and runner bean arrived in Spain and spread across Europe and later arrived in Africa (Gepts and Bliss, 1988; De Ron et al., 2015, 2016a). Since these species were originated and domesticated in tropical highlands, local widely different biotic and physiological abiotic conditions and farmer preferences and/or initial genetic bottleneck had a strong influence on the development of European landraces (Rodiño et al., 2006; Rodriguez et al., 2013; Raggi et al., 2014; Ferreyra et al., 2017) which resulted in a complex genetic structure of the bean germplasm and in a clear differentiation of the European gene pool with respect to the American genetic pools (Santalla et al., 2002; Angioi et al., 2010; Spataro et al., 2011; Rodriguez et al., 2013).

As for common bean in particular, no records of this crop have been found in European herbariums earlier than 1543; but, according to Zeven (1997), common bean was certainly widely grown in many areas of Europe in 1669. Of the many landraces found across Europe most belong to the Andean gene pool, being less represented the Mesoamerican gene pool (Rodiño et al., 2001; Angioi et al., 2010; Leitão et al., 2017). However, a relatively high proportion of the European common bean germplasm (33–44%) appear to be derived from hybridization events between the Andean and Mesoamerican gene pools, when the landraces were grown in proximity, displaying novel genetic combinations not typical of the primary American centers of domestication and emphasizing the potential value of the European germplasm for breeding (Santalla et al., 2002; Angioi et al., 2010).

## Eco-Physiological Adaptive Traits of Bean and Cowpea in the South of Europe

The evolution of beans and cowpea in the South of Europe by their adaptation to the ecophysiological regional conditions has involved changes in landraces. In a study that included 10 cowpea landraces from the South of Europe (five from Portugal, three from Spain, and two from Greece) cultivated in three different locations for 2 years, Martos-Fuentes et al. (2017) displayed the existence of significant interactions among genotypes, locations and years, that is relevant in breeding for important agro-morpho-physiological traits.

The diversity originated in cowpea along centuries is also important for the tolerance to local stresses that each local variety has developed due to natural selection for adaptation as well as farmer selection for agronomical applications and dietary benefits (De Luca et al., 2018).

### Low Temperature

A desirable characteristic for crops is a rapid and homogeneous seed germination and emergence at different environmental conditions (Revilla et al., 2005). As other crops, there are differences among bean landraces regarding their performance (germination, seedling emergence, vegetative growth, flowering, and yield) in different environments and under different temperatures. There is a need of bean germplasm with the qualities of the grain demanded by consumers to increase the success and the added value of the bean crop; and the tolerance to low temperature after sowing at germination and emergence is a key characteristic for a good development of the crop. The eco-physiological response of beans to low temperature stress has been often studied under controlled environmental conditions in glasshouse and climatic chambers but the long-term main goal of genetic improvement for low temperature tolerance is the selection of landraces under different environmental temperatures in open field.

To analyze the response to a relevant eco-physiological condition as low temperature, De Ron et al. (2016b) performed several trials with 28 dry bean genotypes (21 landraces from Spain and Portugal and seven improved varieties from Spain, France and Japan) in open field under different temperature conditions in April (low temperature: 10–14°C), May (moderate temperature: 12–17°C) and June (warm temperature: 15–22°C) in the North of Spain. The experiment was replicated in a growth chamber resembling the same environmental conditions. Three Spanish landraces (PHA399, PHA419, and PHA1058) and the improved variety Borlotto with low temperature stress-tolerance at seedling emergence, and high yield potential could be valuable genetic material for breeding programs. Seedling emergence of the large seeded landraces from Spain belonging to the Andean genetic pool was delayed compared to the small seeded landraces from the Mesoamerican one, in the controlled growth chamber and in the open field experiments, and they showed lower emergence in the open field under realistic agronomic conditions. This fact could be explained by the evolution of the common bean in the southwest of Europe, since farmers probably selected for years large seeded bean landraces due to their high market value and used to germinate the seeds in nurseries before transplanting the seedling into the open field and no breeding actions were taken by farmers and breeders to improve germination and emergence of the large-seeded Andean landraces under low temperatures in field.

The runner bean frequently requires moderate or warm temperatures for a good emergence and growth, while low temperature at sowing delays plant emergence and early growth, and can reduce establishment of the crop when an early sowing is carried out. Rodiño et al. (2007a) evaluated runner bean germplasm in a climate-chamber: 19 landraces from Spain and Portugal, four from Mexico, four from Rwanda and five commercial varieties. Best performers in emergence and first trifoliate leaf, traits related to earliness, were four Spanish and one Portuguese landraces compared to the Mexican ones that indicated a good adaptation of this genetic material to the eco-physiological conditions in the South of Europe.

Cowpea is considered a cold susceptible crop; however, cowpea is not being improved for cold tolerance. Modern breeding programs establish as key breeding objectives the development of drought tolerant, early maturing, pest tolerant and erect type cowpea in countries where this is an important crop, such as India (Roy et al., 2016). Since several years ago, some reports have found genetic diversity for cold tolerance in cowpea, e.g., El-Kholy et al. (1997) evaluated a collection of cowpea genotypes for cold tolerance at germination and found that genotypes differed in rate for leakage of electrolytes but not in maximal percentage of germination. The effects of cold conditions on crop development differ at diverse growth stages, from germination to reproduction. Under cold conditions in the field, reducing the seed moisture content results in decreased percentage of emergence and rapid electrolyte leakage in cowpea, while deep sowing results in slow and low percentage emergence (Ismail et al., 1997).

The genetic regulation of cold tolerance in cowpea has been scarcely studied; but several genes related to cowpea response to cold conditions have been identified (Tan et al., 2016). Ismail et al. (1997) proposed that cold tolerance at early stages of the crop is due to a seed dehydrin protein and can be explained by a single gene inheritance. These authors also reported that maternal gene effects are important for the electrolyte leakage of cowpea seeds at cold temperature, and appear to restrict their contribution to cold tolerance to the beginning of plant development. Although genetic diversity for cold tolerance is limited in cowpea, breeding cowpea for cold tolerance at germination has been successful (Ismail et al., 1997). Although some sources of cold tolerance have been identified, introgression of cold tolerance in elite germplasm is a challenge because cowpea is a mainly self-pollinating crop. Several major QTLs have been identified, even though the development of mapping populations is a difficult and time-consuming task. Markers have not been actually used in breeding; nevertheless, novel techniques, such as developing transgenic plants, RNA-Seq, and reverse genetics open new opportunities for molecular breeding (Tan et al., 2016).

### Water Stress

Water deficit is considered a relevant agronomic factor limiting crop productivity and is responsible for important yield reduction in many crops (Serraj et al., 2004). The severity of drought stress is always unpredictable as it depends on factors such as occurrence and distribution of rainfall, evaporative demands of the atmosphere and moisture storing capacity of the different soils.

In the common bean, the main selection criteria for drought resistance is the plant growth and the grain yield. Rodiño et al. (2007b) evaluated 21 common bean accessions (12 landraces from Spain and Portugal and nine resistant and susceptible cultivars) in two locations in the northwest of Spain to identify those genetic materials adequate for breeding for water deficit tolerance. The Drought Intensity Index (DII) was calculated as DII = 1-Xds/Xns, being Xds and Xns the average of all the accessions under drought stress (DS) and no stress (NS) conditions. Drought Susceptibility Index (DSI) for each common bean accession was calculated following these formulae: DSI = (1-Yds/Yns)/DII, where Yds and Yns are the average yields of an accession under DS and NS conditions. Five Spanish landraces (PHA432, PHA471, PHA543, PHA683, and PHA2074) had high level of drought resistance together with two cultivars (Alavesa and Linex). These results confirm that during its evolution in Europe some common bean landraces were able to adapt to different eco-physiological conditions, such as drought.

Moreover, the variability present in 23 cowpea landraces collected from Greek fields revealed potential germplasm for drought tolerance (Lazaridi et al., 2016). Cowpea is considered a legume tolerant to heat, drought and poor soils due to its capacity to fix nitrogen even under stress conditions (Carvalho et al., 2017a; Lazaridi et al., 2017). However, the diversity available for stress tolerance in southern Europe has neither been deeply studied nor used in breeding programs for stress tolerance. Shadeya et al. (2000) found limited diversity for drought tolerance in advanced breeding lines evaluated by a rapid laboratory method, but they were able to identify tolerant and susceptible genotypes that could be used for breeding. Genetic regulation of drought tolerance follows an additive – dominance model in most crops, including cowpea; being dominance and additive effects similar and important, while epistasis was rare, and narrow sense heritability was low to moderate for most traits under terminal water stress (Olajide and Ilori, 2018).

Drought effects in cowpea included reduction of plant growth, yield components, shoot and seed nutrients, and leaf water content, along with membrane instability; while increase activity of leaf antioxidant enzymes, content of leaf proline, electrolyte leakage, and shoot Si content (Merwad et al., 2018). Furthermore, leaf anatomical features are also altered by drought, being width of midvein and xylem, thickness of midvein, phloem and xylem tissues, and palisade and spongy tissues of leaf blade decreased (Merwad et al., 2018). Drought reduces plant cell water potential and turgor and raises solute concentrations. The water deficit had negative influence on mineral nutrition and metabolism decreases leaf area and alters assimilate partitioning among the organs. Physiological mechanisms of the plants for facing water stress include escape, avoidance by increasing the water uptake and reducing transpiration rate by maintaining tissue turgor by osmotic adjustment allowing the plants to preserve their vegetative growth, and resist the severe water stress by physiological mechanisms (Jones, 2004).

### Biological Nitrogen Fixation

Legume biological Nitrogen (N) fixation by symbiosis with soil rhizobia provides an eco-physiological and agronomic chance to increase common bean productivity related to soil fertility and climatic conditions. Miranda and Bliss (1991) reported that many common bean landraces have low biological N fixation capacity probably due to their original domestication process as a home garden crop, with low selection pressure for the improvement of the symbiotic association with rhizobia. Rodiño et al. (2011) studied 64 common bean landraces for their capacity to establish symbiosis with rhizobia in controlled conditions and the effect of the environmental conditions on the symbiotic efficiency of them in six environments in Spain. The variation among environments for nodulation efficiency among landraces was remarkable, and five of them (PHA175, PHA508, PHA525, PHA595, and PHA652) displayed good nodulation and high yield in field.

The bean-rhizobia symbiotic system is usually affected by the water availability. Coleto et al. (2014) studied the inhibition of N fixation and ureide accumulation under water deficit in two common bean landraces and two breeding lines of contrasting drought tolerance. Their results displayed relevant genotypic differences in the drought sensitivity of biological N fixation among the landraces, and that the genetic variation is linked to ureide accumulation in the stressed leaves. In addition, two common bean landraces studied (PHA246 from Spain and PHA683 from Portugal) had better performance under DS than the tolerant breeding line used as check (Sea 5); therefore, their eco-physiological adaptation were reliable and they could be used in breeding programs designed to improve the efficiency of N biological fixation under water stress in common bean in the South of Europe.

De Ron et al. (2014) evaluated 10 common bean landraces from Spain and Portugal, together with some breeding lines tolerant to water deficit. This material was inoculated with 10 distinct strains of *Rhizobium* (eight local and two checks, *R. tropici* CIAT899 and *R. etli* CFN42) in greenhouse both under irrigation and water stress. Under water stress, five Spanish and one Portuguese landraces displayed high nodule number, high nodular biomass, a great uniformity in the caliber of their nodules, and a significant correlation with aerial biomass that is a relevant component of plant yield.

The results of these experiments showed that the common bean landraces are well adapted to their eco-physiological environments in the South of Europe and some of them are able to establish an efficient symbiosis with native rhizobia, even under water stress conditions.

Low soil fertility is a challenge for cowpea production, especially in low-input agriculture, which is the most common production system in undeveloped countries where this legume is a basic food supply. Fortunately, this crop has a great ability to synthetize N through the symbiotic interaction with rhizobia. Adaptation of cowpea includes coevolution with indigenous rhizobia associated with strains of the species *Ensifer fredii* that were able to nodulate and fix N in cowpea but not in soybean and common bean (Tampakaki et al., 2017). These authors conclude that the *Ensifer* isolates may constitute a new symbiovar for which they propose the name “aegeanense”. Furthermore, symbiosis can partially explain the gains in breeding programs for agronomic performance; actually, Oruru et al. (2018) reported that modern cultivars of cowpea had higher root colonization, nodulation, and nutrients in the shoot than old cultivars and concluded that the response of mycorrhizal inoculation has been indirectly improved by selection for yield.

## Morpho-Agronomic and Genetic Traits of Bean and Cowpea in the South of Europe

In the European Mediterranean basin, clearly differentiated common bean landraces exist, originated from populations firstly introduced in the Iberian Peninsula after the exploration of The Americas. A particular case is the white seed bean types from Turkey that seem to be phylogenetically distant from the rest of the European germplasm, probably due to their introduction through East Asia via the Silk Route (De La Fuente et al., 2010).

There are great differences in the preferences of the bean markets and consumers in different countries and regions related to grain size, shape, color, and cooking time, therefore these types are described as “market classes” (Voysest, 2000; Santalla et al., 2001), usually including unimproved germplasm (landraces) and some improved varieties. Breeding for commercial varieties in beans within landraces of different market classes has the goals of improve the preferred seed size, shape, color, and pattern in each area of production. As mentioned above, in the South of Europe bean landraces appear to have experienced major phenomena of evolution and adaptation, as they show clear differences between them.

In Portugal, the national common bean production still depends considerably on landraces adapted to local conditions, and fulfilling specific morphological, agronomic and nutritional farmers’ preferences in mainland north and central regions, Azores and Madeira Islands (Moreira and Veloso, 2009; Vaz Patto and Araújo, 2016). Currently there are some common bean cultivars (six landraces and two conservation varieties (“Corno de Carneiro” and “Tarrestre”) registered at the Portuguese National Catalog (CNV, 2017). Based on morphological and reproductive traits, considerable diversity has been described among common bean landraces from the North of Portugal (Rodiño et al., 2001) and from Madeira Island (Freitas et al., 2010). In particular, different sources of resistance and partial resistance to rust and powdery mildew have been identified in a dedicated germplasm collection screening (Leitão et al., 2013), anticipating a high potential for disease resistance breeding in the Portuguese germplasm. The genetic variation of the Portuguese common bean accessions was also characterized using RAPD and SSR molecular markers (Martins et al., 2006; Leitão et al., 2017) not detecting clear relation between the geographic distribution and the genetic distance. This absence of relation may be due to an important genetic flow resulting from the traditional seed exchange practices at local markets or among farmers. Leitão et al. (2017) also positioned the Portuguese germplasm in the worldwide diversity of common bean through a SSR-based genetic diversity study involving an enlarged collection of Portuguese landraces from all traditional bean-growing geographical areas and wild relatives and representative accessions from the Andean and Mesoamerican gene pools. Structure analysis divided the collection into three main clusters, with most of the Portuguese accessions clustering with the Andean representatives, but one third of the analyzed national landraces might represent putative hybrids between American gene pools. Some core collections were developed by the same authors maximizing the genetic and morphological diversity of the original collection, and representing the Portuguese common bean accessions with the minimum redundancy.

In Spain many common bean landraces have been collected in farmer fields, starting from the 70’s of the last century and are conserved *ex situ* in the national gene bank (CRF-INIA, Alcalá de Henares) as well as in breeders collections in different regions, while many landraces can still be found cultivated *in situ* (on-farm) in some places mainly for own or local markets consumption. Six areas of Spain have landraces or local varieties awarded with quality labels (PDO and PGI), including 16 common bean and one runner bean, while no cowpea variety is recognized with these labels. The most appreciated landraces or local varieties are white large and extra-large seeded (50–100 g 100 seeds^-1^) (Santalla et al., 2005), generally belonging to the Andean gene pool, although some of them are intermediate or recombinant types with the Mesoamerican pool (Santalla et al., 2002).

In Italy over 200 common bean landraces are officially recorded as maintained *in situ* (Negri et al., 2013) with six of them awarded with PDO or PGI. An analysis of 146 Italian landraces based on the combined use of morpho-physiological, biochemical and molecular traits clearly distinguished almost each landrace from the others (Raggi et al., 2013). It also showed that the Italian landrace genetic diversity is clearly structured in three clusters and that clustering is not simply ascribable to the original Mesoamerican and Andean gene pools, similarly to what was found in the Portuguese germplasm by Leitão et al. (2017). On the contrary, the distinction of cluster 1 from cluster 2 appeared to be (at least partially) due to adaptation (for flowering date and resistance/tolerance to diseases) to the different environmental conditions that are determined by altitude since the presence of selective effects was detected for some of the SSR used in the study. Breeding activities have been intense in the past years since beans are largely cultivated for both the seed and the pod consumption in Italy: twenty-eight cultivars have been released by a Ministry of Agriculture Research Center (CREA_CI, A. Carboni pers. comm.). Most of them were specifically bred for resistance to the main biotic stress of the crop (which are striking on intensive cultivations) and mostly relying on alien germplasm since landraces, although giving product of high organoleptic quality, are poorly adapted to intensive cultivation (Parisi and Campion, 2010). However, to breed lines specifically suited to organic agriculture we can well take advantage of landrace germplasm (Caproni et al., 2018).

In Greece bean landraces have been collected in organized expeditions particularly during the previous century and are conserved *ex situ* in many genebanks while several landraces can still be found cultivated on-farm in many places mainly for own or local consumption. The dry beans of several Greek *Phaseolus* spp. landraces have been characterized as PDO or PGI having added value that resulted in the need for testing the authenticity of their products and the development of test methods based on molecular tools (Ganopoulos et al., 2012). Improved cultivars bred in Greece are either selections from Greek landraces or selections following crosses between landraces and introduced germplasm. Characterization and evaluation of common bean landraces and main improved varieties cultivated in Greece using morpho-agronomical, physicochemical traits, sensory and molecular data showed a wide (among and within landraces) genetic variation and also revealed promising landraces with superior yield components and protein content that could be used “*per se*” or in breeding programs for conventional or low input/organic cultivation (Mavromatis et al., 2010; Vakali et al., 2017).

As for runner beans, different landraces are also cultivated in Spain, Portugal, and Italy (Santalla et al., 2004; Spataro et al., 2011; Rodriguez et al., 2013; Schwember et al., 2017), and in the North of Greece where two groups can be distinguished depending on seed dimensions (“giants” with 100 seed weight range from 120 to 180 g and “elephants” with 100 seed weight outreaches the 180 g).

Cowpea is currently a crop of minor importance for Europe; however, considering its greater drought resistance in comparison with common bean and a scenario of climate change and unpredictability, it is likely to have an increased importance in future years. For instance, Portugal has presently one cowpea cultivar (“Fradel”) registered at the National Catalog for commercial use (CNV, 2017) although many varieties are available commercially. Cowpea cultivation is mostly based on landraces and scientific studies have been carried out to assess breeding potentialities of local germplasm (Negri et al., 2000, 2001; Lazaridi et al., 2016, 2017; Carvalho et al., 2017a,b; Karapanos et al., 2017; Martos-Fuentes et al., 2017). The characterization of fresh pod traits in thirty-one cowpea landraces from Portugal, Spain and Greece revealed promising variation for production (Lazaridi et al., 2017).

From all the above, we may conclude that Southern Europe is still rich in landrace diversity maintained *ex situ* and *in situ* which represents an important source of interesting plant traits combinations, not yet fully explored in formal genetic improvement programs. According to the PGR Genesys (2018) database the landraces of warm season legumes landraces from the South of Europe maintained in genebanks are: 11371 of common bean, 1442 of runner bean and 940 of cowpea. *In situ* wealth of landrace diversity is presently threatened by the replacement by novel, genetically uniform cultivars, the possible general effects of climate change in plant physiology and growth, the aging of farmers and ineffective transmission of knowledge related to landraces, the desertion of the land caused by migration from rural areas to cities, the internationalization of food systems and the pressure of changing markets with restrictive food standards.

However, it should be noted that some of the numerous landraces of warm season legumes were/are being awarded of EU quality marks (27, including both common and runner bean) and/or are promoted as typical product locally. This helps, at least partially, halting the loss of useful germplasm and its evolution *in situ*. The on farm conservation of landraces could be guaranteed if it offers an income to the farmers. This can be achieved by marketing the landraces products emphasizing their uniqueness with special brand names that highlight their local cultural heritage (Karanikolas et al., 2017). Additionally, *in situ* conservation can be accomplished by supporting farmers willing to cultivate the traditional varieties, for example with a participatory plant breeding program, or even with financial support when the genetic resources are considered as national patrimony.

## Concluding Remarks

According to the available data, the variability of the common bean, runner bean and cowpea landraces from the South of Europe is adequately preserved *ex situ* in germplasm banks and in breeders collections in Portugal, Spain, Italy and Greece (Table 1); however, on-farm or *in situ* conservation in isolated areas mainly in gardens and small fields for farmers own consumption and local markets is not guaranteed currently. In addition, this variability is being used for the genetic improvement of varieties, some of them already registered and others protected by quality labels, despite the reduction in the production and consumption of grain legumes in those areas. Legume research programs in Europe are only focussed to cowpea pre-breeding, even though this crop could make significant contributions to legume production in arid areas.

**Table 1:** Warm season legume landraces *ex situ* collections in the South of Europe.

Country	National collections (gene banks)	Breeder collections and features
**Portugal**	Portuguese Plant Germplasm Bank, BPGV (Braga, Portugal, FAO code PRT001; now conserving also the previous INIAV Research Unit of Biotechnology and Genetic Resources collection, FAO code PRT005). Conserving 3307 common bean and 344 cowpea accessions.	
**Spain**	National Center for Plant Generic Resources (CRF-INIA, FAO code ESP004). Conserving 3616 Common bean, 121 runner bean and 487 cowpea accessions.	MBG-CSIC (FAO code ESP009) (De Ron et al., 2018). Conserving 1701 Spanish and other origins common bean, 49 Spanish runner bean and 89 Spanish and Portuguese cowpea accessions (De Ron et al., 2003).
**Italy**	UNIPG-DSA3 (FAO code ITA363) and other Italian collections (See https://www.crea.gov.it/853/plantares/ for details on number of conserved accessions).	Common bean 552, runner bean 91 and cowpea 16 accessions.
**Greece**	Greek Genebank (FAO code GRC005). Conserving 436 common bean, 30 runner bean 30 and 37 cowpea accessions (Katsiotis et al., 2009).	

The genetic structure of landrace populations, in the case of autogamous species such as common bean and cowpea, gives an opportunity for individual selection within landraces adapted to particular eco-physiological conditions with the objective of obtaining improved breeding lines that could be used *per se* for production or as basic germplasm for breeding programs. In the case of the runner bean, an allogamous species, individual selection must include isolation because of the role of insects in the reproductive process.

To take full advantage of these valuable bean and cowpea adapted landrace resources it is extremely important to complement the existing molecular/morpho-physiological diversity analysis with detailed phenotypic evaluations, and to enhance the symbiotic system legume-rhizobia for an efficient biological N fixation. These will allow the identification of landraces with increased market value (adapted to biotic and physiological stresses or characterized by market quality traits) that can actively be used to overcome different constraints affecting both production and consumption. It will result in obtaining environmental-friendly improved legumes for a sustainable production in the South of Europe and for other regions of the World.

## Author Contributions

ADR, PB, VN, MVP, and PR equally contributed to the conception of the work, revising the work, and approval of the version to be published.

## Conflict of Interest Statement

The authors declare that the research was conducted in the absence of any commercial or financial relationships that could be construed as a potential conflict of interest.
